# Sustainable Implementation of Digital Assistive Technologies in Health Care Through a Simplified Interaction and Control Platform: Protocol for a Cocreative Feasibility Study

**DOI:** 10.2196/63089

**Published:** 2025-03-18

**Authors:** Pascal Müller, Sebastian Hofstetter, Patrick Jahn

**Affiliations:** 1 Health Service Research Working Group, Acute Care, Department of Internal Medicine Faculty of Medicine, University Medicine Halle (Saale) Martin-Luther-University Halle-Wittenberg Halle (Saale) Germany

**Keywords:** digital assistive technologies, human-technology interaction, mixed methods, cocreation, user-centered design, health care, intention to use, feasibility study, long-term care

## Abstract

**Background:**

With the expected increase in the number of people needing care and the increasing shortage of skilled care workers, new care concepts are required. Therefore, digital assistive technologies (DATs), especially robotics, can improve the situation of people with different needs and create opportunities for participation. For a human-technology interaction to have a high level of usability, DAT’s meaningfulness and effectiveness must be accessible to end users. Significant barriers to the use of DATs in health care are the lack of controllability and adaptivity, as well as control functions that are too complex.

**Objective:**

The objective of this paper is to develop an interaction and control platform that is understandable to a layperson and has a programming interface for DAT interactions. The innovation consists of the expansion of usage and interaction options for carers of existing DAT in a more individual manner. This is to be achieved by combining modern interactive media, a modular software architecture, and already available DAT.

**Methods:**

The project is planned as a mixed methods study with a longitudinal design, with multiple user involvements and measurement times in collaboration with 3 care facilities in Germany. When assessing technologies, the satisfaction of the basic human needs of competence, connection, and autonomy plays an important role in the actual use of the technology. These needs can be measured in the form of usability (System Usability Scale), intention to use (Technology Usage Inventory), and satisfaction with the carers’ needs (Technology-Based Experience of Need Satisfaction). In the qualitative assessment, user experience is recorded using the think-aloud method and focus groups in order to obtain information about potential improvements of the platform.

**Results:**

The EduXBot (Educational Exploration Robot Application Platform) project was initiated in January 2023 and is scheduled to conclude in December 2025, at which point the project’s final results are expected to be available. The initial results were attained in the summer of 2024 when the final concept for the platform prototype was developed. In November 2024, an initial prototype of a functional platform for the simplified interaction and control of DAT was evaluated.

**Conclusions:**

It is expected that the open DAT system architecture enables caregivers without any previous technical knowledge to assemble their individual DAT functional portfolio. The results of the project will provide low-threshold access to interaction options for existing DAT as well as expand the usage of such technologies in an individual and patient-centered way.

**Trial Registration:**

Deutsches Register Klinischer Studien DRKS00034195; https://drks.de/search/de/trial/DRKS00034195

**International Registered Report Identifier (IRRID):**

DERR1-10.2196/63089

## Introduction

Digital assistive technologies (DATs) have long been discussed as a way to address pressing health care challenges. On the one hand, these challenges are related to the aging of society and the associated increasing need for care services. On the other hand, the acute and future shortage of nurses makes it difficult to provide care for older adults and those who are sick [1-3]. The digital transformation of health care and the use of DAT could be an opportunity to address these challenges [4]. As the health care system in Germany is under pressure to respond to increasing care needs, one measure is to promote the digital transformation of the health care system [5-7]. However, preliminary work shows that DAT, such as mobile apps, telemedicine systems, and robotics, have not yet been implemented in care processes as sustainably as expected [8,9]. However, when used correctly, DAT can provide opportunities to reduce the burden on caregivers [10-13].

The term assistive technology is a generic term for assistive, adaptive, and rehabilitative devices, which, according to the World Health Organization [14], includes all assistive devices, such as crutches, bedpans, or wheelchairs. For the purposes of this study, the term is expanded to include digital technologies such as augmented or virtual reality technologies or robotic systems. A generally valid definition of DAT is difficult to formulate, however, because DAT can develop potential in different areas [15-17], the effects of which would then have to be demonstrated in a specific application.

Nursing can be described as a complex situation in which the patient must be approached individually and according to the situation. The advent of DAT presents a challenge to health care professionals, who must adapt these technologies to the diverse and individual needs of the patients they care for and integrate them into care processes that must be planned individually. Thanks to their training and professional experience, professional nurses are able to quickly understand these complex situations and make appropriate decisions. It is difficult for nurses to imagine that an algorithm-based system can learn their rules and principles and have the flexibility to know when to modify those rules or not apply them at all. Apart from this, nurses recognize that DAT could reduce the error rate for routine tasks that require high concentration, such as medication preparation or documentation [18].

In addition, ethical concerns play an important role in nurses’ reluctance to use DAT. An aspect of the discussion is the change in the workflow that occurs with the integration of DAT. Nurses fear that the logic of the devices, which is different from their own, will cause additional stress or even make them feel influenced by others. Nurses also fear losing their jobs as technology takes over their activities. Last but not least, there is a fear of losing interpersonal contacts. A change in the psychosocial component of nursing work goes hand in hand with a reduction in the attractiveness of the profession for nurses and should, therefore, be avoided [18,19].

Previous work shows that technology development research has been approached from a technology-centered perspective. Reference to user interests is often only made in the context of raising awareness among target groups, identifying needs in the testing phase, or for a finished technology [20-22]. Participatory design approaches such as cocreation are a solution to foster usability and user acceptance. Cocreation is defined as a collaborative approach that involves end users and relevant stakeholders in all phases of a project [23]. Early involvement of end users can increase acceptance and have a positive impact on patient satisfaction and quality of care [24,25]. In addition, cocreation can increase the successful implementation of evidence-based interventions and policies through equal and deep involvement of end users [26,27].

In order for DAT to unfold its potential, it is necessary, on the one hand, to provide services that are tailored to the respective functional limitations of those in need of care so that the use of DAT can be planned and problem oriented [28,29]. This means that nurses also play a crucial role in the widespread use of DAT. According to a study by the Bertelsmann Foundation, the acquisition of knowledge about existing technical systems is a prerequisite for the use of DAT in care settings, as is the development of application skills on the part of nursing staff [30]. Preliminary work also shows that application knowledge and skills, as well as the opportunity to use DAT in relation to care problems, are beneficial for nurses’ willingness to use DAT [31-34].

To fill this research gap, the EduXBot (Educational Exploration Robot Application Platform) project aims to develop and evaluate a technology-based interaction platform with a reduced complexity control interface as part of a cocreative, exploratory approach to facilitate the control of existing DAT by caregivers. The goal is to provide formal caregivers with a simplified way to use DAT to support caregiving. The aim of this feasibility study is to investigate to what extent a prototype for simplified control of already available DAT changes the willingness of nurses to use them in the nursing process. The collaborative project brings together developers from the field of technology research, nursing scientists, and business partners from the fields of project management for digital work environments and the development of digital formats for knowledge transfer. The cocreative nature of the project means that in addition to the scientific project staff, nurses will be involved as end users in every phase of the project. This ensures that the project goals are better achieved in terms of user needs and technical feasibility.

## Methods

### Conceptual Framework

The project aims at the participatory development and evaluation of a platform to promote the applicability of DAT in care settings. A feasibility study with a longitudinal design will be conducted based on a mixed methods approach. The main goal of the platform is a simplified application of DAT to support care processes. The development will be carried out in a participatory manner as a user-centered design (UCD) based on the suggestions of the “Motivation, Engagement, Thriving in User Experience” (METUX) model [35], which takes into account the expectations and experiences of the end users and promotes the intention to use (ITU) and thus the usability, relevance, and creativity of the platform, thus concretizing the overall implementation concept.

In practical implementation, the project is based on the theoretical model of UCD and follows the suggestions of the cocreative design cycle [36]. The UCD approach is used to cocreatively develop a more needs-based and more tailored end product based on the nurses’ expert knowledge and to take possible consequences of implementation into account [36-38]. Positive effects are promised in terms of improving the identified outcomes as well as greater usability and user acceptance of technical products [39,40]. The cocreative design cycle is the process by which all relevant stakeholders are involved in the design and development of technologies. End users are actively involved in the design and development of solutions that meet their challenges. The result of the process is innovative and creative solutions that are useful, effective, and user centered, thereby promoting their actual use [41].

**Figure 1:**
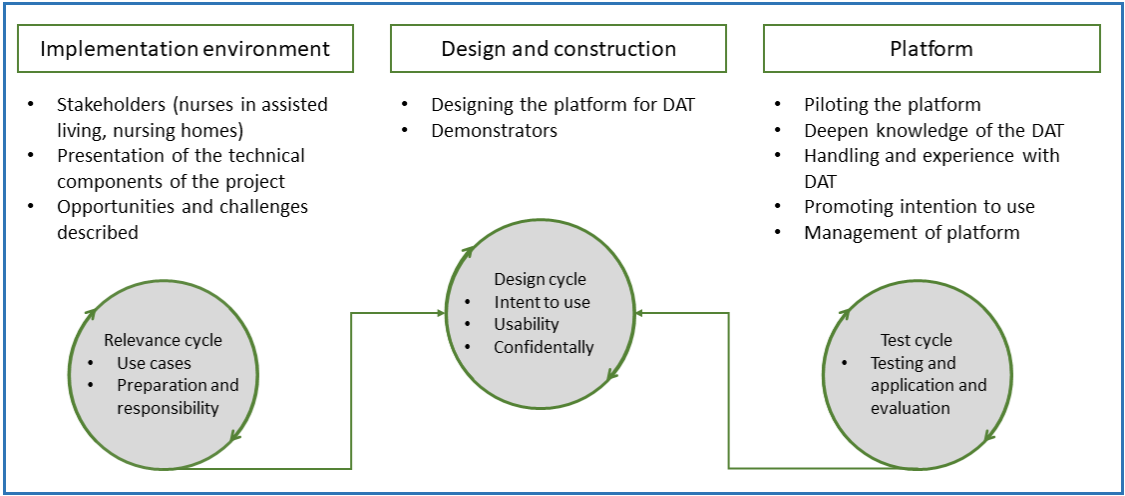
User-centered and cocreative design of the platform development (according to Farao et al [36]). DAT: digital assistive technology.

The user-centered and cocreative approach used in this study integrates 3 cycles (Figure 1) that incorporate the realities of end users (relevance cycle) and the scientific knowledge base (rigor cycle) into the development of technical products (design cycle) [42-45]. With this chosen approach, it is better possible to theorize, collect, and ultimately practically map the requirements for the technologies used in the study in the sense of determining needs. At the same time, collaboration between scientists and end users is more possible. Furthermore, end users can be more actively involved in product evaluation over the course of the test cycle. This ensures the functionality and, ultimately, the success in terms of improved applicability of a platform.

In the first step (relevance cycle), needs, functionalities, and application scenarios of and with nurses as end users are identified through focus groups. The selection of the DAT used in the project is based on the conditions of the cooperating institutions and the needs of the nursing staff. In order to clearly define nursing problems in the context of the learning scenarios, the concept of nursing need (see §14 Sozialgesetzbuch XI [Social Codebook XI]) is used. Based on this concept, DATs are selected that are already available and ready for use. These should cover a wide range and variety of technologies (from a mobile telepresence system to a complex humanoid robot).

**Figure 2:**
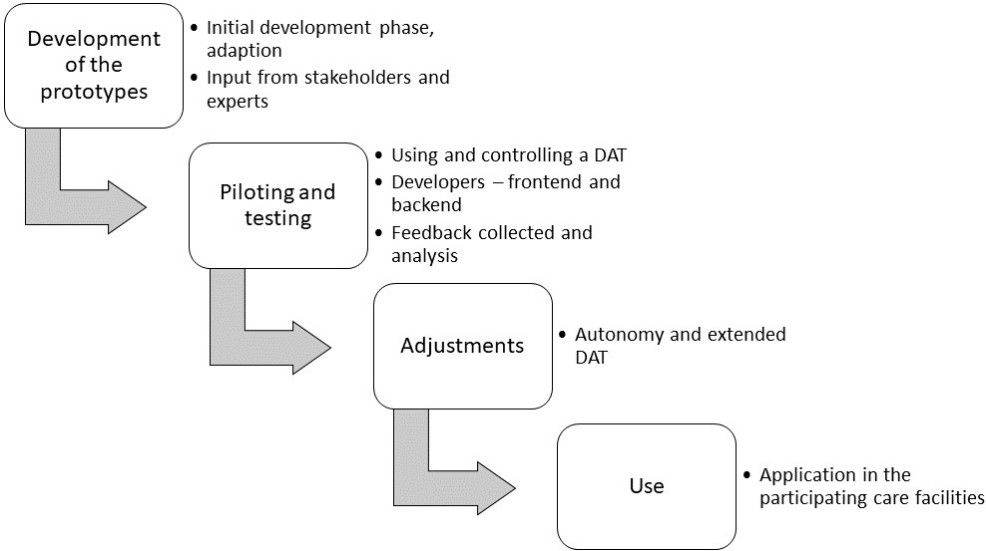
The development of the platform takes place in 4 steps. DAT: digital assistive technology.

Based on the results of this procedure, concrete scenarios will be outlined that are necessary for the implementation of a first demonstration model of the platform. In the next steps, the prototypes will be developed in 4 iterations with respect to usability, user acceptance, and satisfaction of basic psychological needs of the end users until the first prototype can be tested under simulated and real conditions in the facilities. The development of the prototype takes place schematically in 4 steps (Figure 2).

### Technical Description

**Figure 3:**
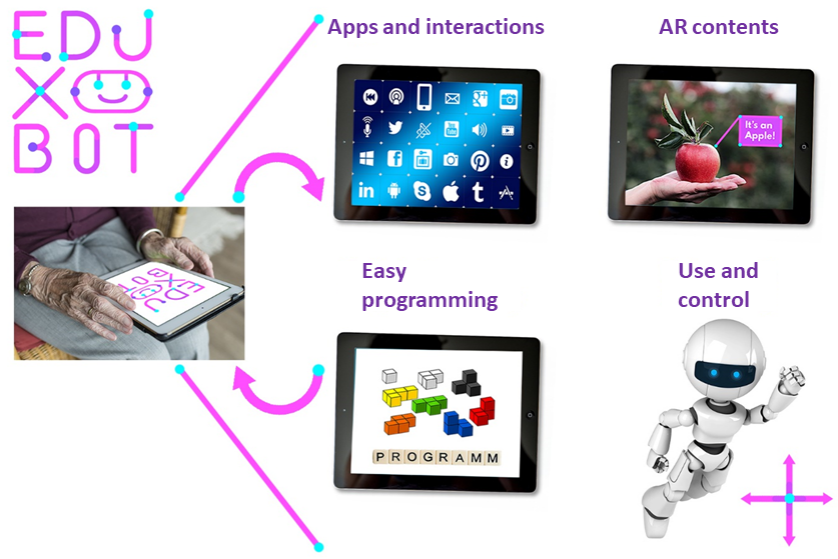
The 4 main functions of the EduXBot platform. AR: augmented reality.

The innovation of the project does not lie in the development of new DAT per se but in the creation of extended usage and interaction possibilities for already available DAT. By combining modern interactive media and a modular software architecture, EduXBot aims to achieve the next level of DAT use in the care of people with disabilities, forced isolation, or restricted movement. The DAT should also promote an individual, programmable user interface for caregivers in an easier way to better meet the needs of those in need of care. EduXBot represents an experience and learning platform with 4 main functions (Figure 3): (1) programming (simplest type of programming), (2) community platform (“widget platform,” users can share their apps), (3) using and controlling (easy way to interact, control, and use the DAT), and (4) augmented reality (AR) experience (experience and learn through AR content).

The multipart concept for using the platform then allows people with diverse previous technological knowledge to use DAT. That means EduXBot leads to the development of a layperson’s iteration with the intention of human-technology interactions, which is developed in the form of a cocreative process together with the end users. EduXBot does not focus on the development of new care technologies that replace caregivers per se but rather on creating supportive interaction options that could relieve the burden on caregivers. The flexible and open system architecture of the platform supports this by keeping different DAT, sensors, and interaction systems available for different usage options. With the help of the development of low-threshold digital access options through an intuitive user interface with community and operator connections, informal support and supply networks are also strengthened as they enable participation in social networks.

### Study Design

#### Overview

EduXBot is a longitudinal feasibility study based on a mixed methods approach. The mixed methods approach combines aspects of quantitative and qualitative research in order to examine the research topic from different perspectives and to answer questions that could not be answered by a purely quantitative or purely qualitative study [46]. The researchers initiated the study with a quantitative phase, which was followed by a second qualitative phase that provided a more in-depth explanation of the initial results [47].

A total of 3 care institutions (1 inpatient, 1 outpatient, and 1 life-sustaining) that support people in their health care and daily life were recruited to participate in the project. The institutions were contacted as partner institutions in previous DAT research projects and agreed to participate in this project. Therefore, it can be assumed that the participating nurses are generally aware of the topic of DAT in nursing. The facility managers will be informed in detail about the project and will be asked to act as gatekeepers to motivate the nurses and relatives in their facility to participate in the research project.

Participants are invited to take part in the study as test subjects by the facilities with which they are affiliated. The participants are as varied as possible in terms of age, gender, length of professional experience, and so forth [48]. The inclusion of nurses from diverse backgrounds within the facilities serves to enhance the heterogeneity of the study sample, thereby contributing to the study’s overall value. It should be noted that some participants may be unable to commit to the study due to scheduling conflicts or other commitments, while others may choose to withdraw (Textbox 1). It is essential to emphasize that no financial compensation is provided for participation in the study. However, those who elect to take part receive certain benefits, including complimentary refreshments during the workshop sessions and, upon request, recognition of their institution and name in the project acknowledgments.

**Description of the inclusion and exclusion criteria of the participants.**

**Inclusion criteria:**

- The participants could be nurses
- The participants could be the support staff
- Currently in a facility that belongs to the project’s practice partner
- Sufficient in written and spoken German

**Exclusion criteria:**

- Those who do not want to participate at the beginning or withdraw later

In the context of research involving human participants, it is imperative to consider pragmatic factors such as financial resources and the maximum feasible number of participants in accordance with the guidelines for designing and evaluating feasibility pilot studies [49]. According to the guideline, achieving saturation in a study occurs at 30 participants. Statistical principles suggest that 30 individuals are sufficient to ensure the normality of the sample size [50,51]. Therefore, the objective is to recruit a minimum of 30 caregivers. It is important to acknowledge that the implementation of blinding is not a viable option in this context, as the participants are required to actively engage with the technologies under study.

The primary objective of this study is to design the human-technology interaction between the platform and users in a manner that ensures a high level of usability for all users. The objective is to assess whether the accessible platform alters the intended use of DAT by offering a simplified application option for caregivers. This inquiry seeks to ascertain the potential for DAT to be integrated as a supplementary resource in the planning of individual care processes. The central question guiding this study is whether the EduXBot platform offers an interface that enhances the autonomy of caregivers’ DAT use. The objective is to enhance usability by a minimum of one level over the course of the study. The primary objective is to ascertain the extent to which the platform affects the usability of DAT (System Usability Scale [SUS]), the intention to use it (Technology Usage Inventory [TUI]), and the satisfaction of caregivers’ needs through technology (Technology-Based Experience of Need Satisfaction [TENS]).

The secondary objective is to develop a multilevel benefit concept for EduXBot. This will provide caregivers with different affinities for electronic media, as well as different levels of technical expertise, and will offer them windows of opportunity to use and experience the platform. The objective is to conduct a direct assessment of the EduXBot platform by the participating nurses and to evaluate the platform with the aim of continuously improving the content and application options of the platform. A secondary objective is to record user satisfaction in order to obtain information on potential improvements to the platform through the analysis of structured feedback. The collection of qualitative data will be facilitated through the implementation of think-aloud (TA) and focus group methods. The aim is to empower caregivers to articulate their subjective experiences and perceptions regarding the utilization of EduXBot in an unstructured manner.

The third objective is the long-term evaluation of the EduXBot platform, encompassing not only its readiness for implementation but also its potential for long-term integration, utilization, and management of DAT. A further objective is to examine the interrelationship between the perceived ITU, usability, and user acceptance of the platform, which is currently being developed for the application. The tertiary target variable is the long-term willingness to use DAT in a sustainable manner and to integrate the digital assistive support offering into the existing care provision framework.

The measurement times are based on the development process shown in Figure 2. This allows changes in improving ITU to be measured. Since there are different versions with different application scenarios, especially at TUI, Table 1 is intended to provide an overview of the measurement times and evaluation methods used.

**Table 1:** Different questionnaires are used at different measurement times.

Measurement time	Evaluation method
T0: Identification of functionalities and application scenarios	TUI^a^ original questionnaire (pre-post version) Focus group
T1: Development of the prototypes	TUI II parallel questionnaire (complete version) Focus group
T2: Piloting and testing	TUI II parallel questionnaire (complete version) SUS^b^ TENS-Interface^c^ Focus group
T3: Adjustments	TUI II parallel questionnaire (complete version) SUS TENS-Interface Focus group
T4: Use	TUI II parallel questionnaire (complete version) SUS TENS-Interface Focus group

^a^TUI: Technology Usage Inventory.

^b^SUS: System Usability Scale.

^c^TENS-Interface: Technology-Based Experience of Need Satisfaction–Interface.

#### Quantitative Evaluation

The TUI is a valid measuring instrument that is based on the established Technology Acceptance Model [52] and its further developments [53]. It comprises 9 subscales, encompassing a total of 33 questions. These questions facilitate the estimation of technology use based on technology-specific and psychological factors. Each scale comprises 3-4 items, which are answered using a 7-point Likert scale. The intention to use scale uses a visual analog scale with a length of 10 cm. The subsequent Table 2 offers a comprehensive overview of the TUI scales.

**Table 2:** Description of the Technology Usage Inventory (TUI) scales according to Kothgassner et al [53].

Scale	Description
Curiosity	Curiosity and inquisitiveness of a person regarding a specific technology.
Technology anxiety	Independent of specific technology. Overwhelm, fear of using technology.
Interest	Independent of specific technology. Interest in technology and willingness to obtain information independently.
Usability	Perceived user-friendliness of a specific technology.
Usefulness	Perceived usefulness of a specific technology. Refers to support in everyday life.
Skepticism	Mistrust of a person regarding the use of a specific technology. Assessment of risk, danger, and disadvantages.
Accessibility	Perceived accessibility (in the sense of availability, procurability) of a specific technology.
Immersion	Can only be specified in connection with the corresponding technologies.
Intention to use	Intention to actually use a specific technology.

The internal consistencies of the scales can be rated as good overall (Cronbach α=0.70 to α=0.89). Furthermore, the TUI scales (with the exception of accessibility) have been found to be valid indicators of stress and relaxation based on heart rate and heart rate variability [53]. The wording of the individual questions can be adapted to the specific technology being evaluated, with the exception of the technology anxiety and interest scales. The TUI’s modular design allows for the exclusion of individual scales contingent upon the investigative objective. For this study, for instance, the immersion scale was excluded, as no technology aimed at immersion was used.

This study uses the questionnaire at each measurement time (Table 1). Before the development of the platform, the original TUI questionnaire pre-post version was used to assess the caregivers’ needs, functionalities, and DAT application scenarios. The “pre” version is administered before the introduction of the DAT, with the objective of collecting data on technology usage tendencies. The “post” version is administered after the introduction of the DAT, with the objective of establishing a baseline for measurement. Subsequent to these measurements, the TUI II parallel questionnaire (complete version) will be administered in its entirety. The measurement enables the formulation of statements regarding changes in the ITU.

For the purpose of evaluation, a cumulative value was formulated for each scale. The cumulative value commences at 3 or 4, representing the lowest level of the construct, and, contingent on the number of items, ranges from 21 (3 items) to 28 (4 items) at the highest level. The ITU scale constitutes an exception in this regard. The scale is evaluated by measuring the distance from the right endpoint (full rejection) to the answer at the intersection of the line. The distance in millimeters was ascertained and added to all 3 items. The maximum scale value that can be attained is 300. The interpretation of test values is facilitated by the scale description. A high test value is indicative of a high level of the respective construct. In instances where the data does not conform to a normal distribution, it is advised to use stanines (standardized calibration scale with a minimum of 1 and a maximum of 9) and percentile ranks (relativizing the test characteristic value in relation to the reference population) [53]. Standard tables have been developed for this purpose (Table 3). Consequently, a statement regarding the proportion of participants who attained the same or lower values is attainable.

**Table 3:** Percentage ranks and stanines.

Percentile rank	Stanine	Percentage (%)
0-4	1	4
>4-11	2	7
>11-23	3	12
>23-40	4	17
>40-60	5	20
>60-77	6	17
>77-89	7	12
>89-96	8	7
>96-100	9	4

The SUS assesses the usability of a system as perceived subjectively by the user and is proven to be technology-independent; that is, it can be used for a wide range of systems and technologies [54,55]. The 10 items are divided into 5 positive and 5 negative statements and are each represented on a Likert scale from 0 to 5. The participants’ answers result in the SUS item score, which must then be converted into the SUS overall score (from 0 to 100) [56].

The calculation of the overall SUS score entails the subtraction of 1 from the raw value of all odd items in the initial step, whereas the raw value of 5 is to be subtracted from the raw value of all even items. To illustrate this calculation, consider the following example. If item 1 had a raw score of 4, the calculated score would be 3 (obtained as 4 minus 1). For item 2, if the raw score was 2, the score was 3 (derived as 5 minus 2). Subsequently, the sum of these scores was calculated and multiplied by 2.5 to derive the overall SUS score [56].

The average overall score of all studies (68) can be used to interpret your overall score [55,56]. Bangor et al [57,58] also introduced a rating scale using adjectives and letters analogous to the American school grading system as an aid to interpretation (Table 4).

**Table 4:** Adjective scale of the System Usability Scale (SUS) overall score [54,55].

SUS^a^ overall score	Area of acceptance	Adjective scale
90-100	Acceptable	Best imaginable
80-89	Acceptable	Excellent
68-79	Acceptable	Good
50-67	Marginal	Ok
35-49	Not acceptable	Poor
0-34	Not acceptable	Worst imaginable

^a^SUS: System Usability Scale.

Deci and Ryan’s [59] assertion posits that the use of the platform is contingent upon the satisfaction of specific fundamental human needs, including autonomy, competence, and connectedness [35]. Psychology posits that the more fundamental psychological needs are met through interaction with the system, the more end users engage with technology [60]. The objective of the TENS-Interface questionnaire is to ascertain the extent to which direct interaction with a technology fulfills the fundamental psychological needs for autonomy, competence, and connectedness [35]. The questionnaires, initially developed in English, underwent translation into German under the guidance of a professional linguist for their usage within the project. The translation process was guided by the “Translation Guidelines and Translation Documentation of the European Social Survey (ESS, 2020)” [61] with a focus on maintaining the validity and comprehensibility of the translation.

In the TENS-Interface, the items are each assigned to the basic needs of competence, autonomy, and relatedness. However, they are presented randomly in the questionnaire. The objective is to ascertain the extent to which direct interaction with a technology fulfills the fundamental psychological needs for autonomy, competence, and relatedness [35]. The application is designed to be universally accessible, ensuring autonomy of action, competence in handling, and a connection with the technology facilitated through the EduXBot platform interface. The collected data are then subjected to evaluation according to these objectives.

The descriptive statistics of the quantitative data are presented depending on the distribution, such as mean or median. Categorical data describing the sample are presented as absolute and relative frequencies. In order to answer the main question about the change in ITU, the difference between the measurement times of the individual test subjects, the facilities, and the overall sample is described as an absolute and relative mean difference. Possible further group differences are examined using the parameters “qualification” and “work experience.” IBM SPSS is used as a tool for data management and data analysis.

#### Qualitative Evaluation

The TA methodology is used to qualitatively evaluate the platform. The idea is to ask subjects to express their thoughts and emotions out loud while testing the prototype in order to document them [62,63]. The advantage of this method is that it collects problems with the technology that has been tried by the end users, as retrospective surveys can lead to incomplete information about the problems with a technology. This means that TA protocols are helpful in understanding the thinking strategies of end users [64].

Testing sessions can be conducted on participants’ own equipment or in a controlled environment. In protocols, participants think out loud as they complete a series of predetermined tasks. Participants are asked to say anything that comes to mind as they complete the task. This may include what they see, think, do, and feel. Sessions are often audio and video recorded so that developers can review what participants did and how they responded. Raw data comprise the verbalization of the thoughts, perceptions, and feelings that participants articulate as they complete a defined task. In a formal research protocol, all verbalizations are transcribed and then analyzed. In the context of usability testing, observers are asked to take notes on what participants say and do without trying to interpret their actions and words, and in particular, to note where they encounter difficulties [65].

The focus groups conducted during the iteration loops are partially structured with the help of guidelines Multimedia Appendix 1. The overall goal is to generate specific knowledge to answer the research question during the development process. The main factors that play a role are the adaptation of the selected application scenarios to the respective facilities as well as aspects of control and usability.

The composition of the focus groups is specifically based on theoretically based preselection in the sense of theoretical sampling according to the indicator caregiver [66]. In addition to these criteria, the most important thing is the willingness to talk about the respective needs and needs in the context of the platform development. No further criteria are defined in advance, but after the sample pool has been generated, the composition is determined exactly with regard to the contrasting of the groups. The number of participants in the focus groups is limited to 6 to a maximum of 10 people [67,68]. The selection of queries takes place according to the principle of theoretical sampling; achieving statistical representativeness is not intended [69].

**Figure 4:**
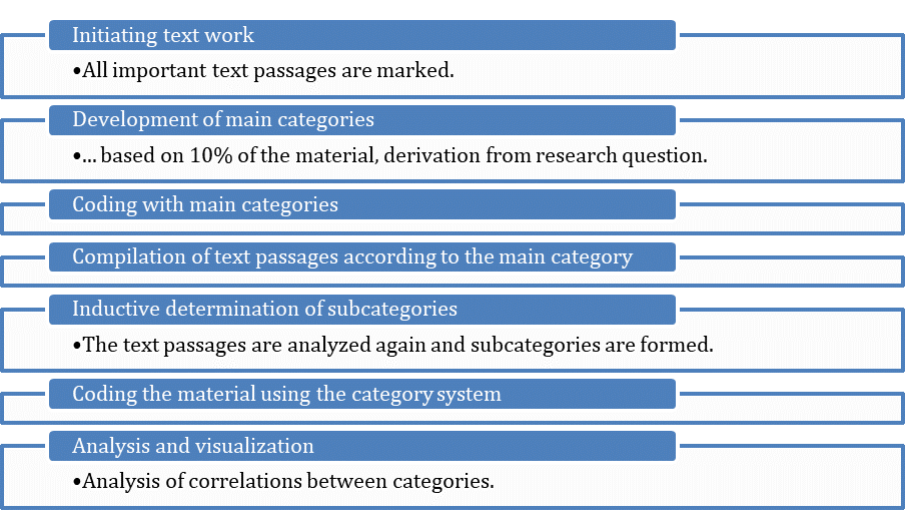
Steps of qualitative content analysis according to Kuckartz and Rädiker [70].

All qualitative data were subjected to Kuckartz and Rädiker’s [70] qualitative content analysis, which provides a structured framework for the content (Figure 4). Given the project’s position within an emerging field of research, the material was coded inductively. This entailed the creation of categories based on the TA protocols and focus groups, thereby facilitating an exploratory evaluation of the material. This methodological approach enabled the systematic organization of the data material according to its content-related characteristics.

### Ethical Considerations

All procedures involving human participants or human tissue will be performed in accordance with the ethical standards and principles of the institutional and national research committee of the 1975 Helsinki Declaration [71] and its later amendments or comparable ethical standards. Informed consent will be obtained from all participants. This study was approved by the Ethics Committee of the Medical Faculty of the Martin Luther University Halle-Wittenberg (approval 2023-190 of August 31, 2023). The study was registered in the German Register of Clinical Studies (DRKS00034195).

## Results

The EduXBot project is funded by the Federal Ministry of Education and Research for a period from January 2023 to December 2025. During the summer of 2023, the practice partners were introduced to the project, and the application scenarios for a prototype design of the platform were cocreatively defined. The initial results were attained in the summer of 2024 when the final concept for the platform prototype was developed in collaboration with the target group. A total of 2 workshops were held in February and July 2024. An initial prototype of a functioning platform for the simplified interaction and control of DAT was evaluated in November 2024. The project is scheduled to conclude in December 2025, at which point the final results will be available. To ensure methodological quality, the Transparent Reporting of Evaluations with Nonrandomized Designs (TREND) statement [72] will be used for reporting the results.

## Discussion

### Principal Findings

The objective of this study is to design the human-technology interaction between an interaction and control platform and nurses in such a way that a process is created that ensures the high usability of DAT in nursing care. The central question guiding this study is whether the low-threshold platform changes the intended use of DAT through the simplified application options for nurses in order to integrate DAT as possible supplementary resources into individual nursing process planning. The objective is to assess whether the platform offers an interface that enhances the autonomy of nurses in using DAT. The evaluation will yield both quantitative and qualitative data, providing insights into the requirements of nurses as end users of DAT. The objective is to enhance the usability of the platform by at least one level over the course of the study.

### Strengths and Limitations

The strengths of this study include the triangulation of quantitative and qualitative data. The potential for DAT to streamline individualized interventions in nursing care has implications for its intended use, which, in turn, affects its actual use in everyday work. The findings of this study will encompass practical, scientific, and societal ramifications, thereby paving the way for subsequent studies and interventions aimed at reducing nurses’ workload. However, the study is not without limitations.

First, the number of participants is limited. For feasibility studies, the Guidelines for Designing and Evaluating Feasibility Pilot Studies [49] recommend basing the number of participants on practical factors such as availability and financial resources. In the event that the targeted number of 30 participants is not attained, the evaluation will be conducted by combining quantitative and qualitative data in accordance with the methods outlined by Creswell and Plano Clark [47].

Second, the participating practice partners have previously engaged in other research projects, have been informed about the digital transformation, and have had experience with DAT. Conversely, participation in this study is voluntary, suggesting that nurses who are generally open to the topic are more likely to participate. The number of participating care facilities is limited to 3, which may limit the generalizability of the findings. While the inclusion of other professional groups, such as support staff, could be considered a valuable addition to the study, future research would benefit from the involvement of a more extensive range of care facilities and health care professionals to enhance the generalizability of the findings.

### Conclusion

A scoping review of the state of the art of robotic interaction and control platforms in health care [73] reveals that only a limited number of feasibility or user studies have addressed the interaction and control of DAT by end users in a cocreative manner. The studies emphasize the necessity of end-user engagement to mitigate ethical concerns and ensure the relevance of the developed technologies to their intended beneficiaries [74-76]. A notable limitation of the existing studies is their exclusive focus on home care settings, resulting in a paucity of empirical findings for the domain of long-term inpatient care. In this context, the EduXBot project has been initiated to address this knowledge gap.

The implementation of technologies in nursing care practice is predicated on nurses’ perception of their meaningful use. The generalization of the care process into standardized procedures is a challenging and complex scenario. The integration of individualized interactions further complicates the scenario, making it challenging for technical developers to meet the requirements and needs of the target group without the involvement of nursing professionals in the development process [77,78]. Caregivers play a pivotal role in research endeavors. Their expertise in the field, stemming from their in-depth understanding of diseases and their impact on patients, positions them as pivotal potential end users. By incorporating their insights, nurses can influence the development of technologies to align with their needs and requirements for interaction and control [79].

The dearth of development expertise among caregivers precludes them from programming the technologies independently. A potential solution to the usability issues of DAT is the development of a platform that provides an interface for nonprogrammers to create individual interventions using everyday controls and a few intuitive steps. This approach has the potential to ensure the sustainable implementation of DAT. EduXBot signifies an inaugural endeavor in this direction.

## Appendix

Multimedia Appendix 1 Focus group guideline (draft).

## Abbreviations

AR: augmented reality
DAT: digital and assistive technology
EduXBot: Educational Exploration Robot Application Platform
ITU: intention to use
METUX: Motivation, Engagement, Thriving in User Experience
SUS: System Usability Scale
TA: think-aloud
TENS: Technology-Based Experience of Need Satisfaction
TREND: Transparent Reporting of Evaluations with Nonrandomized Designs
TUI: Technology Usage Inventory
UCD: user-centered design

## Data Availability

The datasets generated during and/or analyzed during this study will be made publicly available upon completion of the study and can be obtained from the corresponding author on reasonable request.
