# Supplementary material for: Sustainable Implementation of Digital Assistive Technologies in Health Care Through a Simplified Interaction and Control Platform: Protocol for a Cocreative Feasibility Study
Source: JMIR Res Protoc. 2025 Mar 18;14:e63089. doi: 10.2196/63089 (PMC11962334; doi:10.2196/63089)
Supplement: Multimedia Appendix 1 [file resprot_v14i1e63089_app1.docx]

**Guide to the group discussion**

*Narrative stimulus*

“How did you feel about and experience the process of raising awareness of digital assistive technologies (DAT), the evaluation and assessment, the training and the subsequent trial phase here in your facility?”

- Job title or position in the care facility
- Length of time in the profession
- Degree of readiness, objections, etc.
- Concrete framework conditions (location, duration, mode, etc.)
- Comments on the course of the interview
- Comments on the post-interview phase
- Non-verbal information
- Further comments

*Complexes of Questions – Categories*

1. General description of the EduXBot feasibility and the experienced use of technology

- To what extent has EduXBot changed your assessment of DAT?
- How do you rate the different stages of the EduXBot process?
- Does EduXBot facilitate the use of DAT in your nursing work?
- Does it make it easier for you to learn how to use DAT?
- Has EduXBot given you confidence in handling and using DAT?
- Is EduXBot suitable for imparting knowledge and application skills in the use of DAT?
  - If not, what should be different?
  - If yes, why? What makes it different from conventional types of technical instruction?

1. Knowledge reference to DAT (introduction, preparation, specifics of the EduXBot platform)
2. Specification and deepening/reflection of the self-selected scenarios based on the areas of care needs

*Complex of Questions – Conclusion*

- “Are there anything you would like to add?”
- “Do you have any other wishes/suggestions?”
- Thank you!

*Basic implementation instructions*

- Deepening follows immanent basic principle: Identification of problem situations – potential of technical solutions – acquisition of skills and further training modality; guides the discussion using the guidelines; assurance of informed consent
- No rigid arrangement of questions; principle: “openness and specificity” (basic complex sequence should be retained)
- Constant review of degree of abstraction – corresponding flexible deepening
- Immanent before exmanent questions
